# Twitter Improves Influenza Forecasting

**DOI:** 10.1371/currents.outbreaks.90b9ed0f59bae4ccaa683a39865d9117

**Published:** 2014-10-28

**Authors:** Michael J. Paul, Mark Dredze, David Broniatowski

**Affiliations:** Department of Computer Science, Johns Hopkins University, Baltimore, Maryland, USA; Human Language Technology Center of Excellence, Johns Hopkins University, Baltimore, Maryland, USA; Engineering Management and Systems Engineering, The George Washington University, Washington, District of Columbia, USA

## Abstract

Accurate disease forecasts are imperative when preparing for influenza epidemic outbreaks; nevertheless, these forecasts are often limited by the time required to collect new, accurate data. In this paper, we show that data from the microblogging community Twitter significantly improves influenza forecasting. Most prior influenza forecast models are tested against historical influenza-like illness (ILI) data from the U.S. Centers for Disease Control and Prevention (CDC). These data are released with a one-week lag and are often initially inaccurate until the CDC revises them weeks later. Since previous studies utilize the final, revised data in evaluation, their evaluations do not properly determine the effectiveness of forecasting. Our experiments using ILI data available at the time of the forecast show that models incorporating data derived from Twitter can reduce forecasting error by 17-30% over a baseline that only uses historical data. For a given level of accuracy, using Twitter data produces forecasts that are two to four weeks ahead of baseline models. Additionally, we find that models using Twitter data are, on average, better predictors of influenza prevalence than are models using data from Google Flu Trends, the leading web data source.

## Introduction

Accurate disease forecasts are imperative when preparing for influenza epidemic outbreaks. 1 ^,^ 2 This need has driven the research community to bring a multitude of influenza forecasting methods to bear, drawing upon a wide range of statistical techniques and laboratory, clinical, epidemiological, climatological, and demographic data sources. 1 ^,^ 2 ^,^ 3 ^,^ 4 Nevertheless, disease forecasts are often limited by the time required to collect new, accurate data.

Recent work has drawn upon novel web data – especially Twitter 5 ^,^ 6 ^,^ 7 ^,^ 8 ^,^ 9 ^,^ 10 ^,^ 11 ^,^ 12 ^,^ 29 messages and Google search queries 13 – in order to detect influenza rates in real time (i.e., influenza surveillance 14 ^,^ 15 ^,^ 16). Although Google Flu Trends (GFT) has demonstrated some forecast accuracy, 2 ^,^ 17 ^,^ 18 ^,^ 19 it has recently been criticized because of its sensitivity to media reports, the lack of transparency behind GFT data, and the infrequency with which GFT models are updated. 17 ^,^ 20 In contrast, the forecasting potential of open social media, and Twitter in particular, remains largely untested.

In this paper, we demonstrate that influenza surveillance signals from Twitter significantly improve influenza forecasting. We use freely available Twitter data and methods that are insensitive to influence from the mainstream media. 11 ^,^ 23 We are the first to perform an explicit forecast of influenza prevalence rates weeks into the future using social media data, and the first to compare social media to GFT.^28^^,^ 30

Our findings indicate that Twitter data are both more accessible, and provide better forecasts, when compared to GFT data. 17 This is an important validation of social media data sources for influenza surveillance and forecasting.

Our analysis is also the first to use historically accurate surveillance data for the United States. Prior work has relied upon amended data that were not available at the time the forecast was required. These data, consisting of Outpatient Influenza-like Illness Surveillance Network (ILINet) reports from the U.S. Centers for Disease Control and Prevention, are the gold standard for United States influenza surveillance. ILINet data are published with a one-week lag, though some have interpreted the lag as being two weeks, 13 the difference depending the dates used in determining the lag (first day of the reported week vs. the last.) Importantly, the numbers initially released in CDC reports are subject to future revisions as data from additional ILINet sentinel sites arrive. Retrospective analyses of ILINet data generally rely upon the final numbers released by the CDC; not the data initially available. The degree to which updates to ILINet data might impact forecast accuracy has not been previously considered. (The effect of revisions for forecasting in Latin America was recently examined in 28 .) Our results demonstrate that these revisions make a significant difference in forecasting efficacy, further highlighting the benefits of using real-time social media data such as Twitter.

## Methods

**ILINet**

Our baseline surveillance data and gold standard for predictions are based on CDC's ILINet. Historical and current ILINet data (at the national, HHS, and Census-division regional levels) are available from: http://gis.cdc.gov/grasp/fluview/fluportaldashboard.html. Our analyses use the weighted version of this metric, which adjusts for state population. Data are available starting with the 1997-1998 influenza season. Weekly tables released on week **W** of the **X-Y **season are available at the following URLs:

http://www.cdc.gov/flu/weekly/weeklyarchives**X-Y**/data/senAllregt**W**.htm.

In addition to final ILINet values, we downloaded ILINet data that were available at a particular time from these tables. Such tables are available for all seasons beginning 2004-2005.

**** **Twitter**

We use the Twitter influenza surveillance system developed by Lamb et al. 11 ^,^ 23 to produce weekly influenza rates, since it achieves state of the art surveillance results for Twitter data. This algorithm identifies such messages using a cascade of logistic regression classifiers that determine, first, if a message is about health; next, if it is about influenza; and finally, if it is about an influenza infection (rather than simply an awareness of the ongoing flu season). The classifiers are trained purely on the message content rather than on historical ILI data. We used messages from the United States as determined by the *Carmen* geolocation system. 27 We use the output of these models as features for the forecast model based on data from November 27, 2011 through April 5, 2014.

**Google Flu Trends**

Google Flu Trends is an influenza surveillance system that estimates current infection rates based on the volume of Google searches for a select number of influenza related queries. 13 GFT data are available from http://www.google.org/flutrends/us/data.txt. We collected all GFT estimates for the US and restrict our attention to the same time interval as our Twitter data for a direct comparison. Following poor performance during the 2012-13 season, 21 the GFT model was updated in August 2013. Therefore, the numbers beginning in this month are based on the latest model. Retrospective estimates are also available for earlier data using the newest model, but because these estimates are based on a model trained on the same data (in-sample data), they do not provide an accurate assessment of the model's predictive abilities and we therefore do not use them.

**Model**

When forecasting influenza rates, we used a basic linear autoregressive model for ease of comparison to previous work. 17 ^,^ 31 Our linear model took the form:

\begin{equation*}y_{w+k} = \alpha_1 {\tilde{y}}_{w-1} + \alpha_2 {\tilde{y}}_{w-2} + \alpha_3 {\tilde{y}}_{w-3}\end{equation*}

where y_w _denotes the ILI prevalence at week w and the values of a are the regression coefficients. When k=0, we are “nowcasting” – inferring the present influenza prevalence rate that the CDC will report in the following week. 7 When k > 0 we are forecasting further into the future. The \begin{equation*}\alpha \end{equation*} parameters can be estimated using least-squares regression, where parameters are estimated separately for different values of k.

We distinguished between the “gold standard” data that we are trying to estimate – i.e., ILINet data that are no longer being revised – and the data that are actually available on week w-1 from the CDC's weekly reports. Whereas y denotes the weekly value in the final “gold standard” report, \begin{equation*}\tilde{y}\end{equation*} denotes the values that are published in the report that is most recent at the time of the forecast. Our use of \begin{equation*}\tilde{y}\end{equation*} in our model ensures that we more accurately reflect the expected performance of forecasts produced using the most recent ILINet data. When forecasting, we trained our baseline model on three seasons of data, beginning with the 2011-2012 season and evaluated it using cross-fold validation, where each season of data is a cross-validation “fold.” (We also trained with data beginning in 2004, yielding a negligible reduction in error: a decrease of 0.001.)

One advantage of both Twitter and GFT-based systems is that data are available for the current week, not just the previous week. To augment the forecasting model, we include z_w_, the Web-based method's estimate for week w:

\begin{equation*}y_{w+k} =\gamma_1 z_w +  \alpha_1 \tilde{y}_{w-1} + \alpha_2 \tilde{y}_{w-2} + \alpha_3 \tilde{y}_{w-3}\end{equation*}

Similarly, we can utilize multiple Web-based information sources by adding additional terms for each z_w_.

For the purpose of evaluation, we also experimented with a model that uses *only* Web-based data, \begin{equation*}y_w = \gamma z_w\end{equation*}, which fits the web-based data to the corresponding ILINet values.

We include a nonparametric baseline, which predicts each week's value as the average value from all historical data for that week from 1997-2010. That is,

\begin{equation*}y_{w+k} =  \bar{y}_{w+k} = \frac{1}{2010-1997} \sum_{i=1997}^{2010} y_{w+k}^{i}\end{equation*}

where y^i^_w _is the value at week w in the season starting in year i and ending in year i+1.

The purpose of this comparison is to understand how much information we are gaining using autoregressive models over simply modeling each season as the average of previous seasons.

## Results

**Table 1. Mean absolute errors from cross-validation across three seasons for the “nowcasting” task:** (a) Final revised CDC weekly estimates; (b) the realistic model using original CDC data before revision; (c) the model augmented with Twitter data; (d) the model augmented with GFT data; (e) the model augmented with both Twitter and GFT data; (f) values predicted by measuring the historical average.

	Model	11-12	12-13	13-14
(a)	Revised CDC (y)	0.10	0.24	0.24
(b)	Original CDC (~y)	0.20	0.30	0.32
(c)	Twitter (z)	0.33	0.36	0.48
	Twitter (z) + Original CDC (~y)	0.14	0.21	0.21
(d)	GFT (z)	0.35	0.71	0.89
	GFT (z) + Original CDC (~y)	0.20	0.45	0.28
(e)	Twitter + GFT (z)	0.24	0.67	0.62
	Twitter + GFT (z) + Original CDC (~y)	0.15	0.33	0.21
(f)	Historical Average (~y)	0.95	0.87	1.39

We trained several linear autoregressive models on ILINet data from 2011-2013, and explored their ability to correctly forecast the next week’s influenza rate (k=0). We found that a model incorporating Twitter data outperformed an equivalent model relying only upon historical ILINet data (see Figures 1-2). In addition, Table 1 shows that Twitter improves forecasting in all three seasons. In contrast, GFT failed to reduce error in two of the three seasons. Furthermore, adding GFT data to a model that already incorporates ILINet and Twitter data actually reduces performance. (GFT's worst season is 2012-13, likely due to its gross overestimate of the peak influenza rate. 17 ^,^ 21 ) Finally, we find that errors using historically available ILINet data are, on average, 42% higher as compared to CDC’s revised estimates that were not available at the time of the forecast. Twitter forecasts always improve upon those that only use historical data. Moreover, the reduction in error that Twitter provides is substantially understated when using the CDC’s revised estimates rather than the initially reported values. Incorporating Twitter reduces nowcasting error by 29.6% when using the values available at the time of the nowcast, but only reduces error by 6.09% when using the final estimates.

**Table 2. The mean (+/- SD) absolute error of two forecasting models after k weeks.** We compared the baseline model based on previous weeks of CDC ILI data to the best-performing model, that incorporating Twitter data. The third column shows the error reduction over the baseline when using the Twitter model.

k	CDC Only	CDC+Twitter	Reduction
0	0.27 ± 0.06	0.19 ± 0.03	29.6%
1	0.40 ± 0.12	0.29 ± 0.07	27.5%
2	0.49 ± 0.17	0.37 ± 0.08	24.5%
3	0.59 ± 0.22	0.46 ± 0.11	22.0%
4	0.72 ± 0.27	0.55 ± 0.14	25.3%
5	0.83 ± 0.33	0.64 ± 0.17	23.6%
6	0.92 ± 0.39	0.73 ± 0.21	20.7%
7	1.00 ± 0.44	0.80 ± 0.24	20.0%
8	1.06 ± 0.47	0.87 ± 0.27	17.9%
9	1.07 ± 0.48	0.89 ± 0.28	16.8%
10	1.04 ± 0.44	0.83 ± 0.30	20.2%

We next considered the accuracy of forecasting several weeks out (k > 0). Table 2 compares predictions based on only historical ILINet data (the baseline model) to those enhanced with Twitter data, up to 10 weeks into the future. We found that the Twitter model’s error after k weeks closely matches the error of the baseline model after k-1 to k-2 weeks. This means that Twitter data provides up to two additional weeks of forecasting ability for a given accuracy tolerance. When attempting to forecast ten weeks into the future (k=10), the Twitter model displays less error than the baseline model of four weeks prior. The Twitter model outperforms the baseline for all values of k. In contrast, the baseline model outperforms a model using GFT instead of Twitter for all values of k.

**Table 3. Summary of revisions made to CDC ILINet data after k weeks, where k=0 corresponds to the first value reported for a given week.** We measured the mean absolute difference (MAD) and mean difference (MD) between the original forecast and the revision after k weeks. The difference from the week’s previous values after k-1 weeks is also shown.

	Change From Final Report		Change From Previous Report	
**k**	**MAD**	**MD**	**MAD**	**MD**
0	0.137	-0.030	n/a	n/a
1	0.083	-0.002	0.101	0.028
2	0.082	-0.008	0.073	-0.007
3	0.060	0.003	0.050	0.011
4	0.053	-0.001	0.027	-0.004
5	0.048	-0.004	0.020	-0.003

Finally, we measured the extent to which ILINet revisions impact the efficacy of forecasts. In addition, we measured the differences in a given week’s ILINet values for subsequent reports (Table 3). We found that the magnitude of these differences is, on average, 12% of the standard deviation of the final values, with the average error gradually decreasing as more data become available over time. Nevertheless, the most recently available ILINet data values are, on average, inaccurate.

**Nowcasting Errors:**
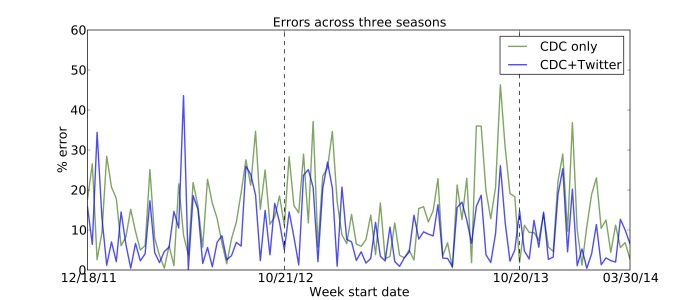
Percent error for three years’ worth of “nowcasts” (forecasts at k=0) using two models: the baseline autoregressive model that uses the previous three weeks of available ILI data (green), and the improved model that adds the Twitter estimate of the current week in addition to the three weeks of ILI values (blue). The vertical lines mark the beginning of a new season. Each season's estimates are based on models trained on the remaining two seasons. The model that includes Twitter data produced better forecasts for 86 out of the 114 weeks shown in the figure.

**Nowcasting Predictions:**
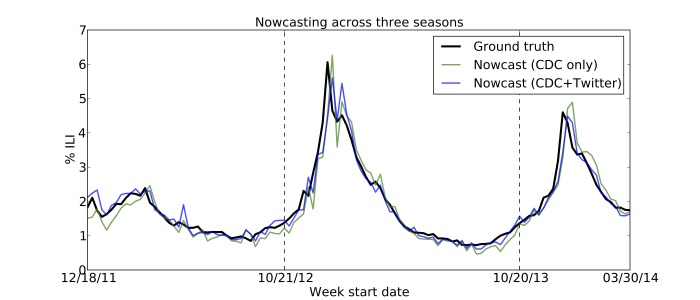
Nowcast predictions for three seasons using two models: the baseline autoregressive model (green), and the improved model that includes Twitter (blue). The ground truth ILI values are shown in black.

## Discussion

Prior work on influenza surveillance using Twitter has not compared results against a simple autoregressive model based on ILINet data. Indeed, we have shown that Twitter data alone are less informative than this baseline model. Our work is therefore novel in that we have established a baseline for comparison against which other models may be tested. We therefore recommend that future influenza surveillance and forecasting methods compare to this simple baseline.

Our analysis is the first to systematically characterize the limitations of ILINet data. In particular, we have found that forecasting studies that use historical ILINet data must account for the fact that these data are often initially inaccurate and undergo frequent revision, effectively increasing the lag between data collection and the time that accurate numbers are available to health professionals. While others have noted the existence of revisions to ILINet, 28 we have shown that these initial measurement errors translate to errors in forecasting. It is here that Twitter and other social media data, which record signals of influenza prevalence in real time, can make the biggest contribution.

Our paper is the first to demonstrate that Twitter data improves influenza forecasts over what can be extracted from non-retrospective ILINet data. Surprisingly, our study found that GFT hurt, rather than helped, forecasts. Previous studies have found that GFT provides better surveillance results when compared to retrospective historic data. 17 ^,^ 22 One possible explanation for this discrepancy is that our study was restricted to only three seasons, during one of which GFT performed worse than usual; however, models trained on additional years of GFT data are not comparable to the Twitter-based models, which were the focus of this study. Other reasons may be that previous studies used revised CDC data or assumed a 2-week lag (instead of the more accurate 1-week lag). As we have argued elsewhere, 26 there are several benefits to using Twitter over GFT, including the ubiquity, openness, public availability, and ease of use of Twitter data. These factors have led the wider academic community to focus on Twitter, especially in light of recent poor performance of GFT, and the attendant concerns about using metrics based on proprietary data and algorithms. 17 As we collect additional years of tweets, we will be able to make broader claims about the relative utility of Google and Twitter data. Furthermore, our results do not preclude new and more sophisticated methods that rely on Google 20 or Twitter data.

While our experiments focused on national influenza prevalence, forecasting systems have much more utility at finer geographic scales. Recent work has demonstrated that Twitter data correlate with ILI rates at the municipal level 23 ^,^ 24 , suggesting that Web data could improve forecasts for cities as well. More sophisticated models are typically used in practice (18, 25), and our encouraging preliminary results motivate the need for experimenting with Twitter and GFT data in richer models, such as those that take full advantage of variables unique to social media (e.g., daily, rather than weekly, ILI estimates).

## Competing Interests

Dr. Dredze reports receipt of compensation for travel for talks at various academic, corporate, and governmental entities and consulting for Directing Medicine, Progeny Systems, and Sickweather. Mr. Paul serves on the advisory board for Sickweather.
